# A scoping review of 24-h movement behaviours research in Chinese children and adolescents

**DOI:** 10.3389/fpubh.2026.1801708

**Published:** 2026-05-12

**Authors:** Zhang Feng, Yuanlong Lou

**Affiliations:** 1 Shanghai University of Sport, Shanghai, China; 2 Hanshan Normal University, Chaozhou City, Guangdong, China

**Keywords:** 24-h movement behaviours, children and adolescents, China, physical activity, scoping review, sedentary behaviour, sleep

## Abstract

**Background:**

Physical activity, sleep, and sedentary behaviour are essential components within the 24-h time frame. A scientific understanding of 24-h movement behaviours is crucial for formulating targeted intervention guidelines and programs. The objective of this study was to map the current research landscape and fill the gaps in 24-h movement behaviours among Chinese children and adolescents.

**Methods:**

Web of Science, PubMed, EBSCO, and, CNKI (China National Knowledge Infrastructure) were systematically searched for relevant studies published between January 2019 and October 2025. The study followed the PRISMA-ScR guidelines, and the literature screening process involved three rounds: duplicate removal, title and abstract screening, and full-text screening. Inclusion criteria: Targeting Chinese children and/or adolescents aged 3–18 years; Focusing on 24-h movement behaviours; Published in Chinese or English; Data extraction included: title, author, year, country, study type, research design, adherence to guideline, sample characteristics, and research results.

**Results:**

Ninety two studies were included in this scoping review. All the included studies were published between 2019 and 2025, showing a generally increasing trend over the years. The review included 817,482 participants aged 3–18 years, predominantly from the general population. Geographically, Shanghai and Guangdong were core regions, while the underdeveloped central and western regions had extremely low representation. Most studies used a cross-sectional design (80), with few longitudinal or intervention ones. For monitoring, device-based tools dominated sedentary behaviours/screen time assessment (31), while questionnaires were the primary tool for physical activity (40), and public data utilization was low. Research variables centered on mental health, covering physical fitness, social interaction, body composition, etc. Fifty-two studies showed average compliance rates of 17.59% (Physical Activity, PA), 51.08% (screen time), 31.62% (sleep), and only 4.91% for all three.

**Conclusion:**

Research on 24-h movement behaviours among Chinese children and adolescents faces multiple challenges, including an uneven geographic distribution of samples, limited diversity in monitoring methods and analytical methods, and low compliance with relevant guidelines. Most studies adopt a cross-sectional design, with few prospective cohort studies and intervention experimental studies. Additionally, there are no guidelines for 24-h movement behaviours tailored to Chinese children and adolescents.

## 1 Introduction

**Figure 1:**
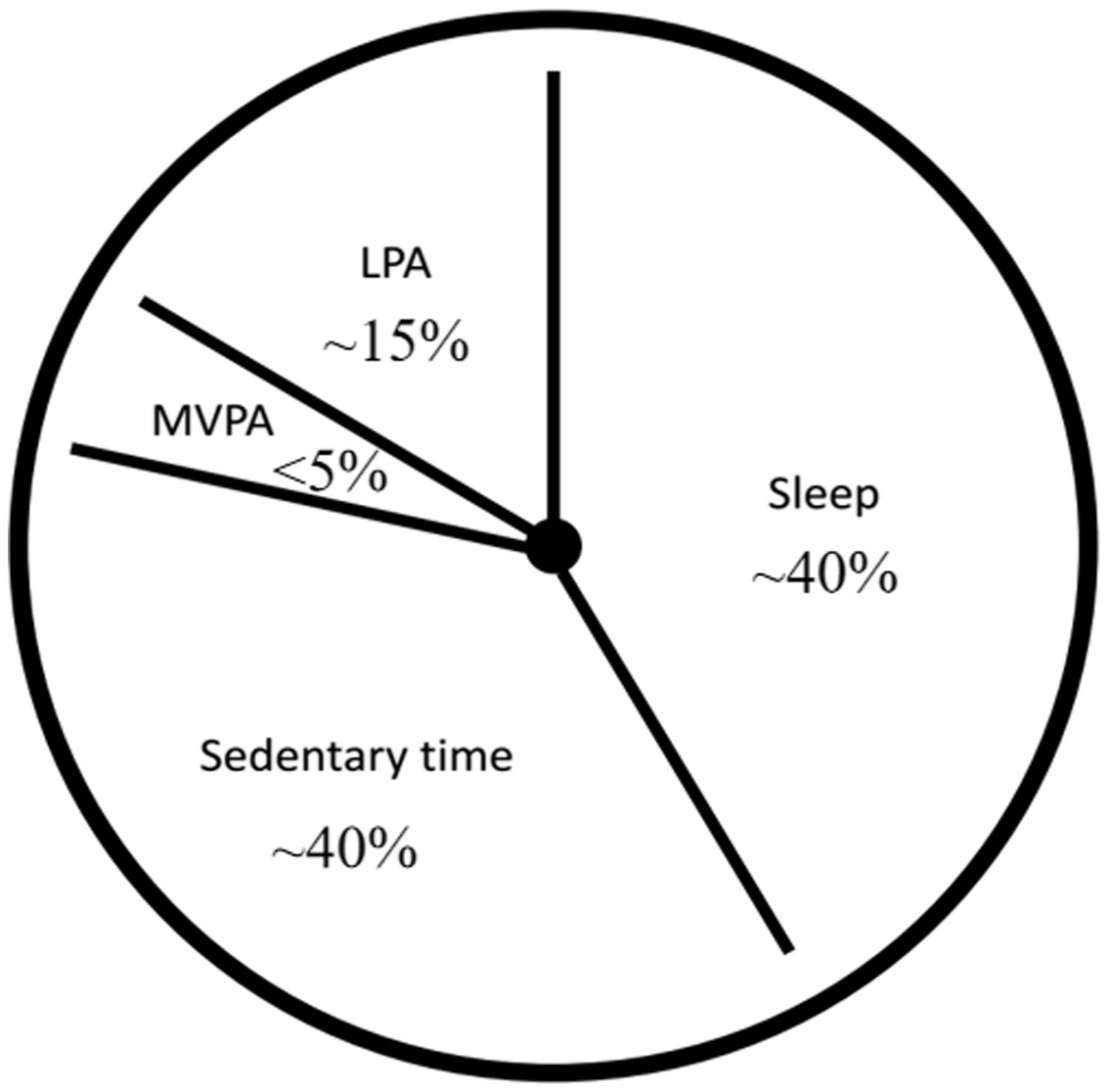
The composition proportion chart of 24-h movement behaviours [adapted from Chaput et al. (1)]. LPA, low-intensity physical activity; MVPA, moderate-to-vigorous physical activity.

The concept of 24-h movement behaviours, proposed by Chaput et al. (1) refers to the integration of physical activity, sedentary time (particularly screen time), and sleep within a 24-h cycle. When studying the impact of physical activity on human health, Chaput argued that although MVPA provides many important health benefits, it accounts for only a small portion of the 24 h (<5%), even among active children and adolescents. In contrast, sleep (~40%), sedentary time (~40%), and low-intensity physical activity (LPA) (~15%) account for approximately 95% of a day (Figure 1). Therefore, Chaput suggested that human 24-h movement behaviours should be regarded as a continuum of movement. Focusing only on MVPA while ignoring other components of the movement continuum limits our understanding of how 24-h movement behaviours interact to affect health outcomes. On this basis, Canada launched the *Canadian 24-h Movement Guidelines for Children and Youth: An Integration of Physical Activity, Sedentary Behaviour, and Sleep* in 2016 (2), and the World Health Organization (WHO) released the *Guidelines on Physical Activity, Sedentary Behaviour and Sleep for Children Under 5 Years of Age* in 2019 (3). The release of these guidelines has promoted a shift in national physical activity behaviour guidelines, from fragmented recommendations focusing solely on physical activity volume to systematic guidelines based on 24-h time-use (4).

Regular physical activity promotes health benefits. Frequent exercise can prevent noncommunicable diseases (5) and is associated with improved mental health (6, 7) and quality of life (8). In contrast, insufficient physical activity increases the risk of poor physical and cognitive function (9), obesity (10), metabolic disorders (11), and mental illnesses (12, 13). A sedentary lifestyle, also known as a sedentary behaviour pattern, refers to a combination of low-energy-consuming behaviours involving sitting or lying. It can also be defined as sitting or reclining postures with an energy expenditure of ≤1.5 METs during non-sleep states, or light physical activity (LIPA) with fewer than 100 steps per minute (14, 15). The WHO *Guidelines on Physical Activity and Sedentary Behaviour* state that excessive sedentary behaviour increases the risk of obesity, reduces cardiorespiratory metabolic function and physical fitness, affects social behaviour, and decreases sleep duration (16). Sleep is an important physiological phenomenon in humans, characterized by the temporary cessation of various conscious active behaviours and reduced responsiveness to external environmental stimuli (17). Insufficient sleep severely impacts mental and physical health as well as the quality of daily life (18–20). Therefore, regular physical activity, reduced sedentary time (especially screen time), and good sleep quality are important guarantees for physical health.

Adolescence is a critical period for individual physical and mental development, and 24-h movement behaviours are closely related to their physical and mental health. Physical activity behaviours and habits have a significant impact on adult behaviours, while adverse health outcomes in adulthood can be traced back to childhood (21, 22). Therefore, systematic research on the 24-h movement behaviours of children and adolescents is of great significance for promoting healthy development in adulthood and improving the quality of life in old age.

Based on the above, this study conducts a scoping review of studies focusing on Chinese children and adolescents aged 3–18 years, with 24-h movement behaviours as the research theme. It aims to summarize the current research status of 24-h movement behaviours among Chinese children and adolescents, identify existing research gaps, propose targeted suggestions for future research, and provide theoretical support for promoting the healthy development of 24-h movement behaviours in this population.

## 2 Materials and methods

### 2.1 Literature sources and search strategy

This research conducted a comprehensive search of published papers in four electronic databases (PubMed, EBSCO, CNKI and Web of Science). Adhering to the preferred reporting items for systematic reviews and meta-analyses extension for scoping reviews (PRISMA-ScR) guidelines, the PRISMA-ScR checklist is provided in Additional file 1 (23). According to Huang et al. (24), research on the 24-h movement behaviours of the Chinese population began in 2019. Therefore, the time frame for literature retrieval was set to 2019 or later. In addition, since 24-h movement behaviour is classified as compositional data (25), our inclusion criteria must cover all three modules: physical activity, sedentary behaviour, and sleep.

The search strategy for each database included search terms in four main categories: population (child, children, youth, adolescent, teenager, juvenile, preschool children); physical activity (physical activity, exercise, movement behaviour); sedentary behaviour [sedentary behaviour (SB), sedentary time, sedentary lifestyle, sitting time, screen time (ST)]; sleep (sleep duration, sleep quality, sleep time). The detailed search strategies are shown in Additional file 2.

Duplicate records were removed using the built-in deduplication function in reference management software prior to systematic screening. Following deduplication, the remaining records were screened against pre-specified inclusion and exclusion criteria.

### 2.2 Inclusion criteria and selection process

Following deduplication and title/abstract screening, full-text articles were assessed against the following inclusion criteria: (1) involved children and/or adolescents aged 3–18 years residing in China; (2) studies focusing on 24-h movement behaviours, including all three domains: physical activity, sedentary time/screen time, and sleep; (3) peer-reviewed original research articles published in either Chinese or English. Studies were excluded if they focused on Chinese children or adolescents residing outside China, or if they were literature reviews, commentaries, conference abstracts, editorials, or case reports.

### 2.3 Data extraction

Data were extracted from the included articles into standardized tables by two reviewers (Zhang and Su) independently. After data extraction, a third reviewer (Zhao) randomly sampled 10% of the data to verify consistency. A Kappa coefficient was used to assess inter-rater reliability, and the result showed a kappa value of 0.89 (95% confidence interval: 0.83–0.95), indicating high consistency between the two extractors. Any discrepancies identified during the verification process were resolved through group discussion until a consensus was reached. The inclusion form contained the following information: title, author, year, country, study type, research design, guideline adherence, sample characteristics (age, gender, sample size), measures of PA, SB/ST, Sleep, and the research results.

### 2.4 Data synthesis and statistical analysis

Descriptive statistics were used to summarize the research landscape (publication trends, geographic distribution, study designs, measurement methods). In this study, a sample size-weighted average method was used to calculate the overall compliance rate with the 24-h movement guidelines, aiming to eliminate biases caused by differences in sample sizes across studies. Specifically, the total sample size of all included studies was first calculated. Subsequently, the proportion of each individual study’s sample size relative to the total sample size was used as the weight. Finally, the weighted average compliance rates for MVPA, sedentary behaviour, sleep, and simultaneous compliance with all three recommendations were obtained by summing the products of “compliance rate of each study × corresponding weight.”

## 3 Results

**Figure 2:**
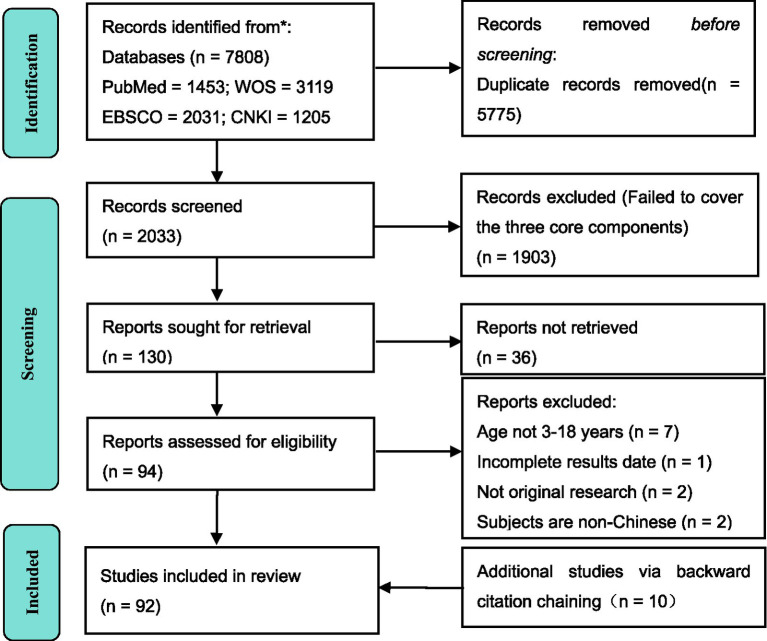
PRISMA flow diagram of the study selection process.

A total of 7,808 studies were retrieved from four electronic databases (PubMed, WOS, EBSCO, and CNKI). After duplicate removal, 2,033 studies were retained for title and abstract screening. Of these, 1,903 were excluded for failing to meet the eligibility criteria. Subsequently, 130 studies were retained for inclusion in this study after a full-text eligibility assessment. Additionally, an additional 10 citations were identified through backward citation chaining and these citations were also included. As such, 92 studies met the criteria for inclusion in this scoping review (Figure 2). The detailed information of the included studies is shown in Additional file 3, and the 92 included articles will not be elaborated on in the reference section.

### 3.1 Analysis of research characteristics on 24-h movement behaviours of children and adolescents in China

#### 3.1.1 Number of published papers on 24-h movement behaviours research

**Figure 3:**
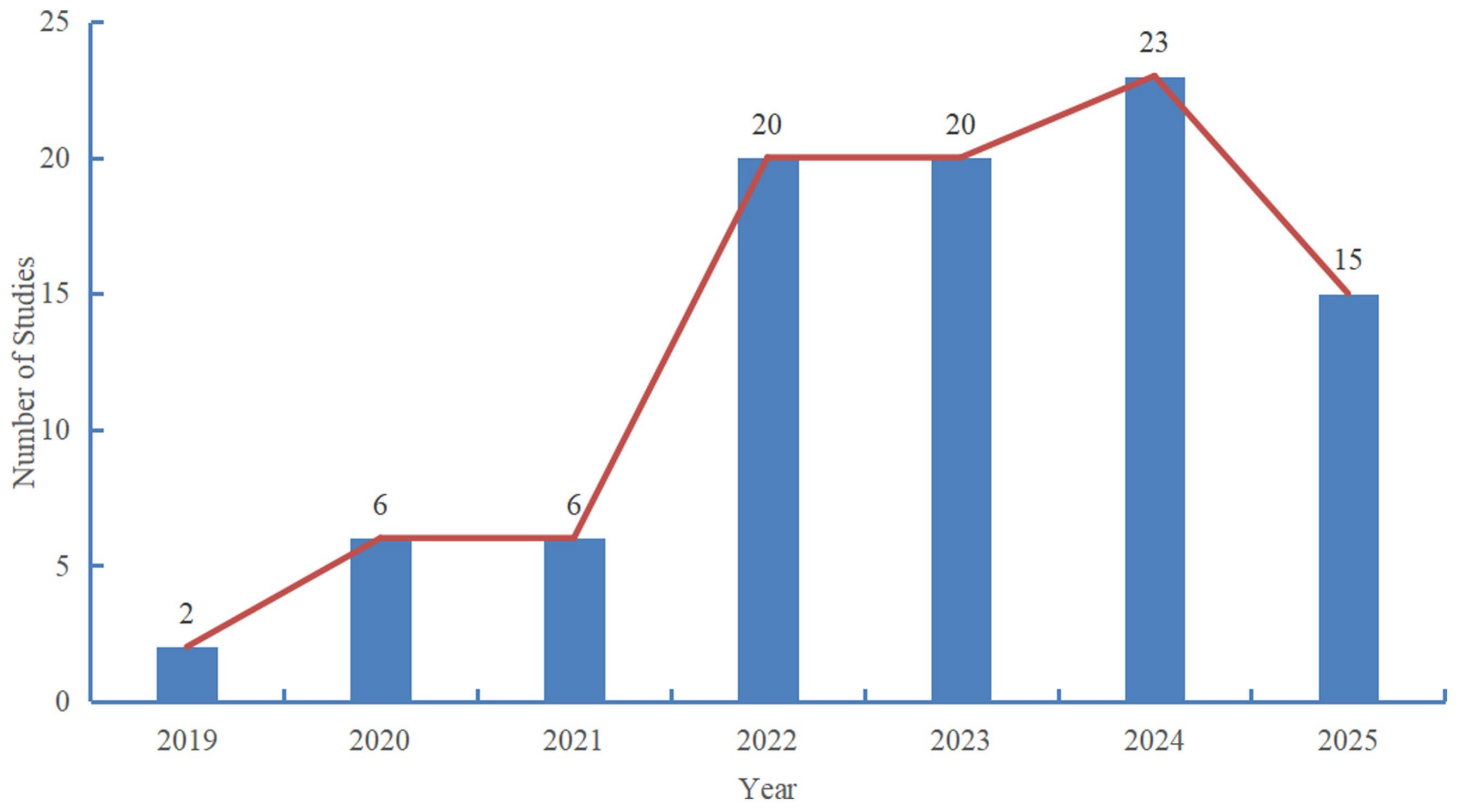
Yearly publication number of relevant research.

As shown in Figure 3, the number of studies on 24-h movement behaviours among Chinese children and adolescents increased steadily from 2019 to 2025. Specifically, 2 papers were published in 2019, followed by an increase to 6 in 2020 and 2021; thereafter, rapid growth emerged in 2022 and remained stable over the subsequent 3 years, with no significant fluctuations observed.

#### 3.1.2 Socio-demographic characteristics of the study participants

As shown in Table 1, the total sample size of this study was 817,482, with boys accounting for 52%. The age range of the sample spanned 3 to 18 years, with 20 studies focusing on preschoolers (3–6 years) and 70 studies targeting school-age children and adolescents (7–18 years).

**Table 1:** Characteristics of the study sample.

Category type	Number/percentage	Category type
Sample size		817,482
Proportion of boys		52%
Years	3–6	20
	7–18	70
General or with condition	General	84
	Autism spectrum disorders	2
	Intellectual disabilities	2
	Overweight and obese children	1
	Lowand middle-income families	1

Regarding the study population classification (general population or populations with specific conditions), 84 studies were conducted on the general population. Among the condition-specific groups, two studies each focused on children with autism spectrum disorders and intellectual disabilities, respectively, while 1 study targeted overweight and obese children, and 1 study selected children from low- and middle-income families as participants.

**Figure 4:**
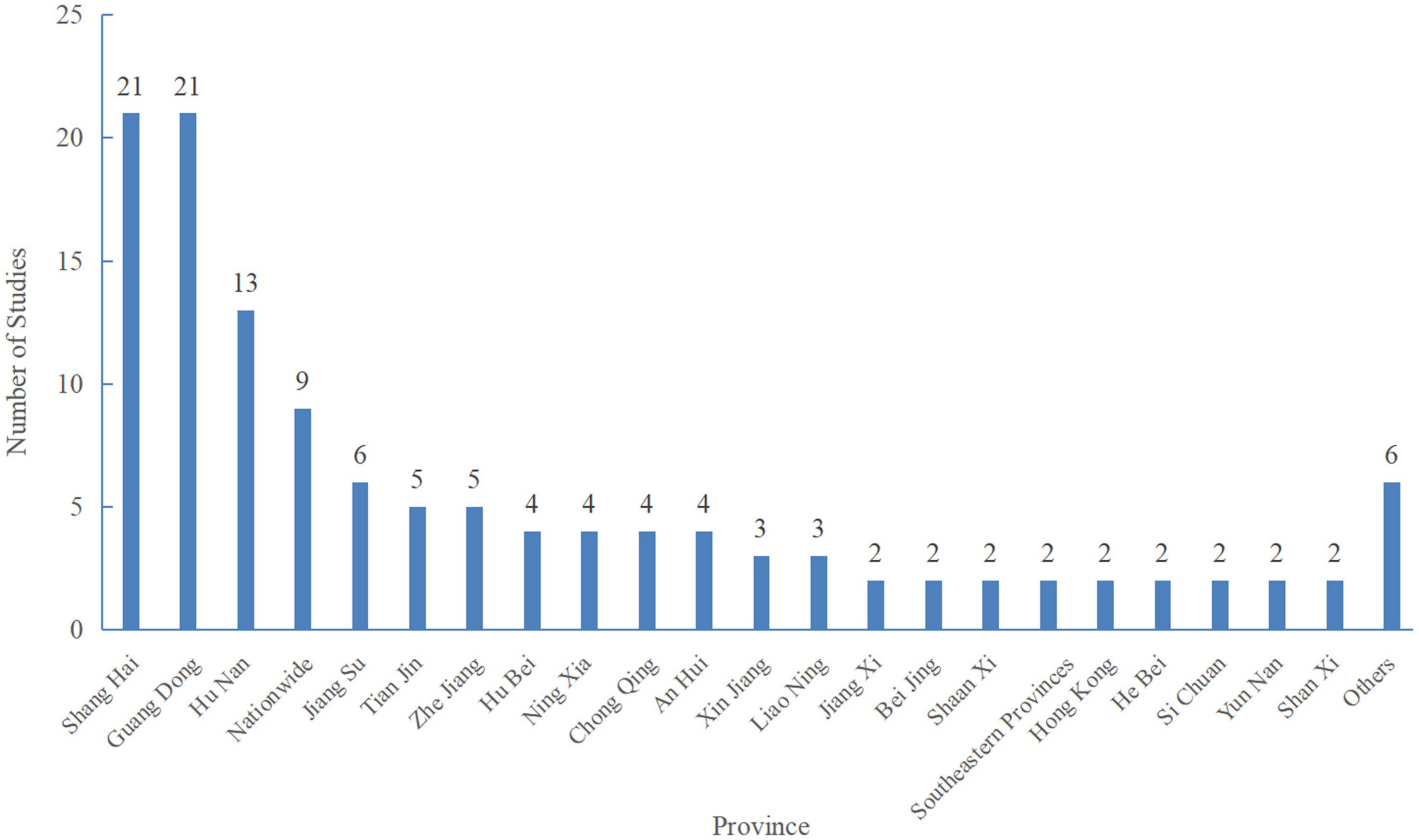
Regional map of 24-h movement behaviours of children and adolescents in China.

In terms of the geographical distribution characteristics of the research subjects, there was a significant regional imbalance (Figure 4). Shanghai (21 studies) and Guangdong (21 studies) were the core regions with the most concentrated research; eastern coastal areas such as Tianjin (5 studies), Jiangsu (6 studies), and Zhejiang (5 studies) constituted the main scope of research coverage. In contrast, only 2 studies were conducted in many regions, including Shanxi, Yunnan, Sichuan, Hebei, and Hong Kong, 3 in Xinjiang, and 4 each in Anhui, Chongqing, Ningxia, and Hubei. The overall proportion of research in underdeveloped central and western regions (e.g., Tibet and Guizhou) remained extremely low. In addition, nine studies were conducted on a nationwide scale, but no comparisons were made between different regions.

(Note: The nationwide studies (*n* = 9) are independent samples and are not included in regional studies; there is no overlapping counting between regional studies and nationwide studies).

#### 3.1.3 Research design and monitoring methods

**Figure 5:**
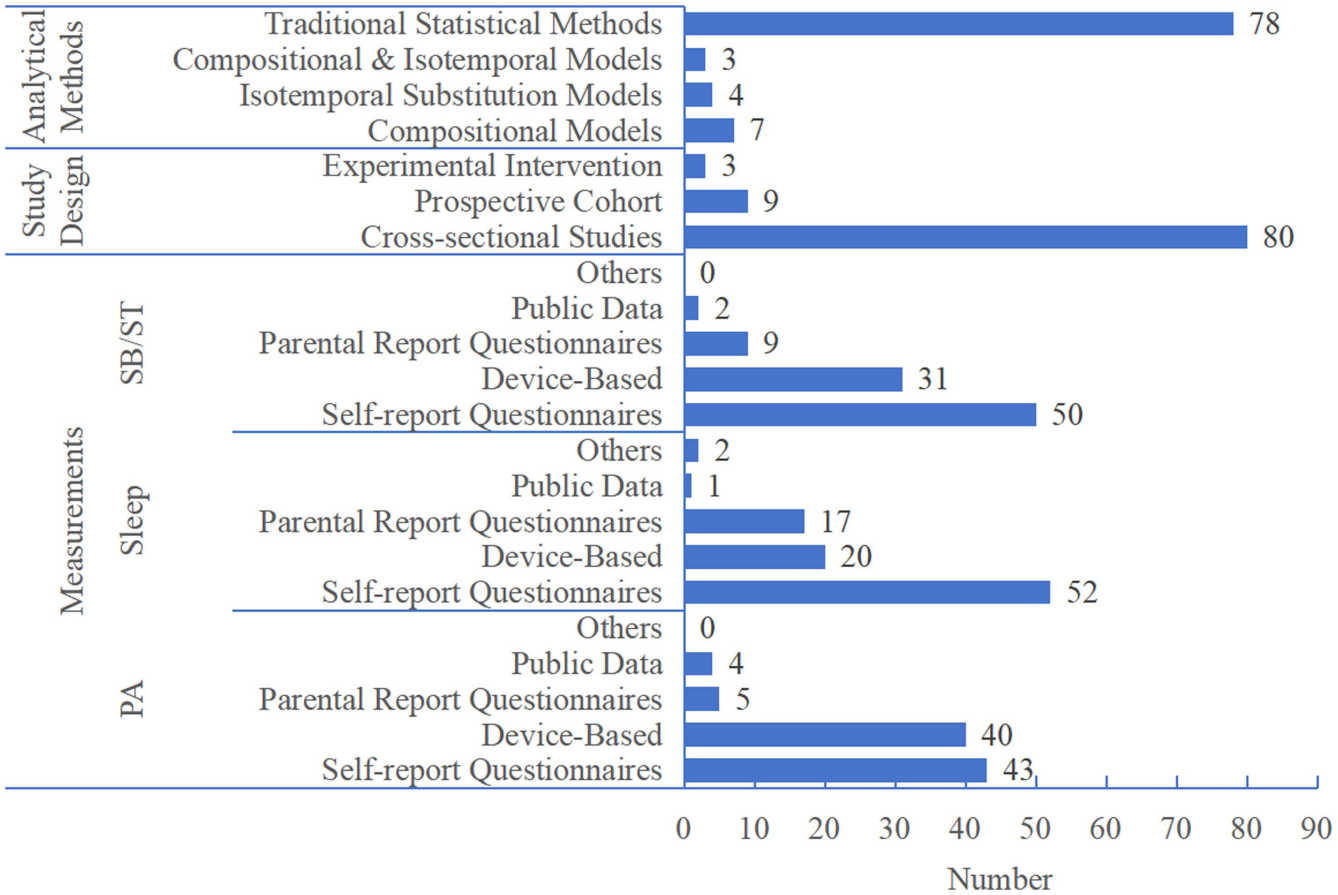
Distribution of study design, measurements, and analytical methods in included studies. PA, physical activity; SB/ST, sedentary behaviour/screen time.

To further clarify the methodological characteristics of existing studies, we analyzed the analytical methods, research design, and monitoring tools adopted in the included literature (Figure 5). Regarding analytical frameworks, traditional statistical methods were applied in 78 of the 92 included studies, while 14 studies adopted advanced compositional analysis approaches. Specifically, 7 studies used Compositional Models, 4 used Isotemporal Substitution Models, and 3 applied combined Compositional & Isotemporal Models. All of these 14 studies were cross-sectional designs, focusing on the associations between 24-h movement behaviour composition and health outcomes such as body composition and physical fitness.

In terms of measurement methods, device-based monitoring was the most widely used in the SB/ST field (used in 31 studies), followed by sleep research (used in 20 studies); In contrast, in PA research, self-administered questionnaires were the primary measurement tool (used in 40 studies). Most sleep-related studies adopted parent-reported questionnaires (used in 17 studies), and the use of public data(*China Education Panel Survey*, *CEPS 2014–2015*, *China Health and Nutrition Survey*)was relatively limited across all fields (SB/ST: 2; sleep: 1; PA: 4). Regarding study design, the existing evidence mainly came from cross-sectional studies (used in 80 studies), with relatively fewer prospective cohort studies (used in 9 studies) and experimental intervention studies (used in 3 studies).

#### 3.1.4 Research variables

Based on Additional file 4, the research variables of included studies were categorized into five core health domains, with a strong focus on physical and mental health outcomes. The findings revealed a strong concentration on health outcomes, encompassing physical health, mental health, and psychosocial development. The proportion of studies focusing on each domain was as follows: mental health was the most extensively investigated outcome, accounting for 73.9% (*n* = 68) of the included studies. It encompassed subdimensions such as emotional problems (e.g., depression, anxiety disorders), conduct problems, behavioural problems, as well as positive psychology factors like life satisfaction and subjective well-being. Physical ability and physical fitness ranked second, representing 67.4% (*n* = 62). This domain included key components such as cardiorespiratory endurance, sprint and agility, muscle strength, flexibility and balance, and fundamental movement skills (e.g., standing long jump, rope skipping). Body Fat & BMI constituted a critical physical health indicator, with 59.8% (*n* = 55) of studies examining this domain. Specific metrics included BMI, body fat percentage (BF%), and waist-to-hip ratio (WHR). Cognitive function studies made up 33.7% (*n* = 31), focusing on executive function (e.g., working memory, inhibitory control), academic-related outcomes, and metabolic indicators. Social Function accounted for 30.4% (*n* = 28), covering social relationships, prosocial behaviours, and problem behaviours (e.g., peer interaction issues).

Additionally, smaller domains such as quality of life and chronic disease collectively occupied a minor proportion of the included studies. Overall, this distribution highlights that current research on 24-h movement behaviours among Chinese children and adolescents heavily prioritizes mental health, physical fitness, and body composition, while relatively fewer studies explore cognitive and social functional outcomes.

#### 3.1.5 Compliance rate with the 24-h movement behaviours guidelines for children and adolescents

Among the 92 included studies, 52 investigated compliance rates among children and adolescents with 24-h movement behaviour guidelines. Most studies refer to the *Canadian 24-Hour Movement Guidelines*; some studies adopt the WHO *Guidelines on Physical Activity, Sedentary Behaviour and Sleep for Children Under 5 Years of Age* and *China’s Exercise Guidelines for Preschool Children (3–6 Years Old)*.

**Figure 6:**
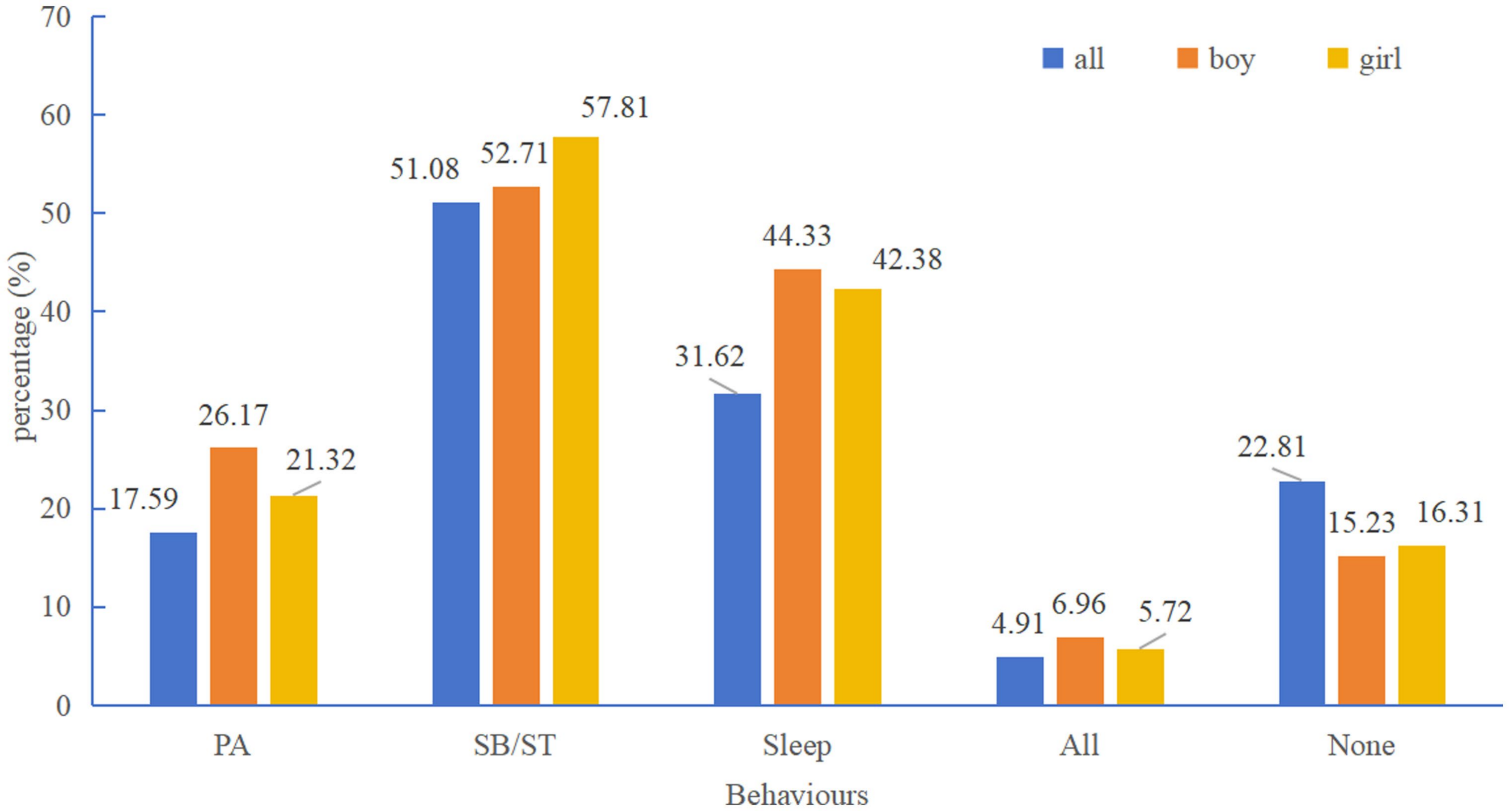
Compliance status with the 24-h movement guidelines. PA, physical activity; SB/ST, sedentary behaviour/screen time.

From the perspective of quantitative analysis, the compliance rate of PA among Chinese children and adolescents ranged from 0.4% to 88.4% (mean: 17.59%), the compliance rate of screen time (ST) ranged from 2% to 93.6% (mean: 51.08%), and the compliance rate of sleep duration (SLP) ranged from 0% to 85.9% (mean: 31.62%). The compliance rate for meeting all three criteria ranged from 0.1% to 33.51% (mean: 4.91%), while the rate of failing to meet all three criteria ranged from 2.2% to 57.2% (mean: 22.81%). As shown in Figure 6.

In terms of gender, among the 15 studies comparing gender, 9 showed that boys’ compliance rates were higher than girls’, 4 found no significant differences, and 2 showed that girls’ compliance rates were higher than boys’. In addition, existing studies have not found differences between regions.

All included studies consistently found that meeting the standards for multiple indicators of 24-h movement behaviours is associated with improvements in various health outcomes among children and adolescents, including weight status, cardiovascular health, psychological well-being, cognitive development, quality of life, and dietary patterns.

## 4 Discussion

Scoping reviews are typically used to map the existing literature in a specific field, outline the nature, characteristics and volume of the literature, and clarify conceptual boundaries to identify research gaps. When the body of literature is large, complex, or heterogeneous, the scoping review method plays a crucial role—it helps identify gaps and determine priority areas for future research, policy, and practice (26, 27). The objective of this scoping review was to map the extensive literature, describe recent research progress and outline the overall characteristics of 24-h movement behaviour among Chinese children and adolescents, thereby providing a reference for subsequent studies.

### 4.1 Current status of 24-h movement behaviours research in Chinese children and adolescents

In terms of the number of published studies, Chinese scholars’ research on children and adolescents’ 24-h movement behaviours began in 2019, and the number of such studies experienced a process of rapid growth followed by stabilization in the subsequent years. Although the literature search was conducted in October 2025, the number of studies published in 2025 remained substantial. Overall, the research field of 24-h movement behaviours in Chinese children and adolescents has shown a trend of steady development after initial rapid growth, which is also consistent with the developmental characteristics of emerging public health research fields in China (28).

In terms of the geographical distribution of research samples, studies on children and adolescents’ 24-h movement behaviours exhibit a severe imbalance, which is highly correlated with regional economic development levels: due to strong talent attraction and sufficient research funding, the eastern coastal regions have become key research areas; in addition, most scholars mainly conduct research on subjects in local areas of specific provinces or cities, lacking nationwide investigations and tests. China has a vast territory, with significant variations in natural environments and cultural characteristics, leading to differences in children’s and adolescents’ 24-h movement behaviours across regions. Future research should strengthen comparisons of children’s and adolescents’ movement behaviour across different regions to inform targeted intervention plans.

In terms of study design, most of the studies are cross-sectional studies, with a small proportion being prospective cohort studies and intervention experimental studies. This conclusion is highly consistent with those of other studies. A large number of studies, when summarizing the limitations of others’ or their own research, have noted that existing longitudinal studies constitute only a tiny minority, and that most results and conclusions are based on cross-sectional studies, which have inherent limitations. Thus, a substantial number of longitudinal empirical studies are needed to verify the obtained results (29–31). Cross-sectional studies cannot determine the causal relationships between behaviours such as physical activity and sedentary behaviours and physical fitness. Research in this field should strengthen long-term follow-up to eliminate biases in research results caused by individual differences, thereby thoroughly exploring the prospective association between the two.

### 4.2 Study designs, monitoring methods and analytical frameworks

Scientific and reasonable monitoring of children and adolescents’ 24-h movement behaviours is a critical prerequisite for targeted interventions in subsequent stages. Currently, the internationally applicable methods for 24-h movement behaviours monitoring are mainly divided into subjective methods (questionnaires, interviews, activity diaries, and direct observation) and objective methods (accelerometers, pedometers).

Our findings indicate that most studies have used self-report methods to collect data on children’s and adolescents’ 24-h movement behaviours, followed by wearable device methods. In addition, for younger participants, researchers have used parent-proxy questionnaires to collect data, and some studies have directly obtained relevant data from public databases.

The differences among the aforementioned monitoring methods reflect the contradictions and challenges in data acquisition in current research in this field: for example, self-reported questionnaires facilitate large-scale data collection but are prone to recall bias. This bias may lead to data distortion and reduce the accuracy of research conclusions (32, 33); parent-proxy questionnaires are prone to interference from parents’ cognitive limitations and subjective judgments, making it challenging to reflect the real movement behaviours of children and adolescents accurately; while studies relying on public data can obtain relatively comprehensive and long-term data, the pertinence and timeliness of such data may be insufficient, failing to meet the needs of specific research closely. Future research could explore integrating artificial intelligence, wearable devices, and the Internet of Things (IoT) to develop novel, cost-effective, high-precision monitoring tools and to construct a multi-source data-fusion monitoring system. This will enable more scientific, dynamic, and comprehensive tracking of children and adolescents’ 24-h movement behaviours, thereby providing more reliable data to support subsequent theoretical research and practical interventions.

The predominance of cross-sectional studies in research on Chinese children and adolescents’ 24-h movement behaviours stems from multiple practical and cognitive factors in the domestic research landscape. First, most research funding is short-term and project-based, with few special funds for long-term follow-up, leaving researchers unable to cover the high costs of sample tracking, repeated data collection and long-term device maintenance for prospective cohort or intervention studies. Second, following up child and adolescent samples poses unique practical challenges: this group experiences rapid life transitions with frequent changes in schools and residences, leading to high sample attrition rates; additionally, securing continuous guardian consent and coordinating with schools for follow-up further increase the operational complexity of longitudinal research. Third, in the initial stage of this research field in China, there is a certain academic cognitive bias: most researchers tend to adopt cross-sectional designs—with their shorter cycles, simpler implementation and quicker results—to identify basic associations between 24-h movement behaviours and health outcomes, while lacking sufficient awareness of the necessity of longitudinal research for exploring causal relationships and long-term health effects (24). Collectively, these factors have led to an imbalance in study design types in this field, hindering in-depth exploration of the causal mechanisms linking 24-h movement behaviours to children and adolescents’ physical and mental health. To address this imbalance, it is necessary to establish special funding for long-term follow-up studies, construct a multi-center research collaboration network to reduce sample attrition, and strengthen academic training to improve researchers’ awareness of longitudinal study design.

The limited application of compositional data analysis (CoDA) and time-use epidemiology frameworks is a notable methodological gap in domestic research. While these approaches have become the international gold standard for addressing the compositional nature of 24-h movement behaviours, only 15% of included studies adopted them. The majority of studies relied on traditional statistical methods, which may introduce bias by ignoring the 24-h time constraint and co-dependence between physical activity, sedentary behaviour and sleep. The 14 included compositional studies were limited to cross-sectional designs and basic model applications, highlighting the need for more prospective cohort and intervention studies using CoDA to clarify causal relationships between movement behaviour composition and long-term health outcomes.

### 4.3 Compliance with 24-h movement behaviours guidelines among children and adolescents

Efforts were made to explore the differences in guideline compliance rates across different age groups (preschoolers: 3–6 years, school-age children: 7–12 years, adolescents: 13–18 years), which are critical for identifying high-risk populations and formulating targeted intervention strategies. However, reliable quantitative comparison and stratified analysis across age groups were not feasible due to two key data limitations of the included studies. First, the age reporting of the 92 studies was highly heterogeneous, with three different reporting formats: mean ± standard deviation (e.g., 5.04 ± 0.88 years), broad age ranges spanning multiple developmental stages (e.g., 11–17 years), and narrow age bands for specific populations (e.g., 14–15 years), with no unified age stratification standard across studies. Second, the vast majority of studies (*n* = 85, 92.4%) only reported the aggregated compliance rate of the full sample, and did not provide original stratified data by age subgroups, including age-specific compliance rates, sample size distribution, and corresponding health outcome statistics. Therefore, for the present study, we were unable to determine the differences in 24-h movement behaviour compliance across different age groups.

The compliance rate with the 24-h movement behaviours guidelines is closely related to the physical and mental health of children and adolescents (34). The results of this study indicate that the overall compliance rate with 24-h movement behaviour guidelines among Chinese children and adolescents is relatively low, which is highly consistent with the findings of numerous domestic and international studies. In her research review focusing on adolescents aged 5–18 years, Wang (35) pointed out that the compliance with 24-h movement behaviour guidelines among children and adolescents worldwide is not optimistic—only 6.85% of the participants in her study met the standards for all three indicators (physical activity, sedentary behaviour, and sleep), with physical activity having the lowest compliance rate, followed by sedentary behaviour. A cross-sectional study by Friel et al. (36) based on data from the U.S. Child Health Survey, also found that only 8.8% of American children and adolescents fully met the three requirements of the 24-h movement guidelines. Although the sleep compliance rate was relatively high (86.0%), the compliance rates for physical activity (23.0%) and sedentary behaviour (32.9%) were significantly lower.

The compliance rates of Chinese children and adolescents show significant gaps compared with international counterparts. A survey by Roman-Viñas et al. (37) on 6,128 children aged 9–11 years across 12 countries further revealed that the compliance rate of Chinese children and adolescents with 24-h movement behaviours is at a low level globally: the overall full compliance rate (2%) is significantly lower than that of Australia (15%) and Canada (14%); the compliance rate for MVPA (15%) is much lower than that of Finland (61%); the sleep duration compliance rate (34.2%) also lags far behind Australia (75.8%); and the proportion of children who failed to meet all three indicators (21.1%) is higher than that of Australia (7%). This cross-sectional comparison clearly highlights the urgency and necessity of intervening in the 24-h movement behaviours of Chinese children and adolescents.

Regarding the relationship between compliance rate and age for children and adolescents’ 24-h movement behaviours, while a study by Chen et al. (38) suggested that compliance rate decreases with increasing age, this study found no differences in compliance rate by gender or region. This discrepancy may be attributed to differences in participants’ ages, living environments, family habits, study designs, and reference standards for guidelines. Future studies should conduct refined comparisons under controlled conditions.

In addition, the compliance rate of Chinese children and adolescents’ 24-h movement behaviours is usually assessed against the Canadian 24-h Movement Guidelines. Currently, China still lacks a scientific and reasonable set of guidelines for 24-h movement behaviours in children and adolescents that covers physical activity, sedentary behaviour, and sleep. Although Chinese scholars issued the first *Physical Activity Guidelines for Children and Adolescents* in 2017, which proposed recommendations on daily physical activity and sedentary behaviour for Chinese children and adolescents (39), the guideline is primarily based on integrating 28 European and American studies (40). It lacks support from localized evidence and does not include sleep guidelines, so its role in standardizing and guiding behaviours is less than satisfactory. Therefore, formulating guidelines for 24-h movement behaviours that align with the actual conditions of Chinese children and adolescents is a key prerequisite for promoting the development of healthy lifestyles.

### 4.4 Strengths and limitations

This study is the first scoping review of 24-h movement behaviours among children and adolescents in China. However, there remains much space for improvement in current research: (1) Owing to language limitations, this study only searched for publicly published literature in Chinese and English, failing to include relevant literature from other countries in this field. This has restricted the comprehensiveness of the literature to a certain extent; (2) The included studies in this review involve a wide range of variables, diverse study types, and varied data analysis methods. As a result, it is impossible to apply methods such as Meta-analysis to conduct quantitative analysis of the study results; (3) Although most studies considered and controlled for some potential confounding factors, the specific confounding factors adjusted and controlled for varied across different studies. This inconsistency may have exerted a certain impact on the study results; (4) Although this review utilized multiple databases (PubMed, EBSCO, CNKI, and Web of Science), searching other databases such as Cochrane Library and PsycInfo may have yielded other relevant published papers relevant to the aims of this scoping review; (5) The age reporting of included studies was highly heterogeneous, leading to the inability to conduct reliable age-stratified analysis of compliance rates with 24-h movement guidelines.

## 5 Prospects

### 5.1 Optimize research layout and design to enhance representativeness and causal inference

Future studies should strengthen sample coverage in underdeveloped central and western regions and conduct nationwide cross-regional comparative research to reduce geographical bias. In terms of research design, it is necessary to significantly increase the proportion of prospective cohort studies and interventional experimental studies. Long-term follow-up can clarify the causal relationship between 24-h movement behaviours and health outcomes, thereby overcoming the limitations of current cross-sectional studies. To ensure the comparability of different research results, the key lies in unifying the definitions of core research variables (such as physical activity intensity and screen time types) and standardizing statistical analysis models.

Methodologically, future studies should systematically adopt compositional data analysis (CoDA) and time-use epidemiology frameworks—including isotemporal substitution models and time-reallocation approaches—to explore the interdependent nature of movement behaviours and the health effects of time redistribution. To facilitate this, researchers should receive specialized training in these advanced methods. Furthermore, the integration of compositional analysis into longitudinal and intervention studies should be prioritized to strengthen causal inference.

### 5.2 Innovate monitoring technologies and methods to improve data objectivity and integration

Efforts should be made to explore integrating artificial intelligence, wearable devices, and Internet of Things technologies to develop low-cost, high-precision monitoring tools and to build a multi-source data fusion system. To this end, reliance on subjective questionnaires should be reduced, and the proportion of objective monitoring should be increased. Meanwhile, the use of public data should be standardized, with clear definitions of its scope and limitations, to enhance data timeliness and relevance. When adopting new technologies and methods, it is essential to establish standardized data quality control procedures, including equipment calibration and standardization of data cleaning algorithms, to ensure data reliability and validity.

### 5.3 Formulate localized guidelines and intervention programs to enhance guidance and applicability

A top priority is to develop exclusive 24-h movement behaviour guidelines for Chinese children and adolescents that account for regional cultural characteristics, lifestyles, and environmental differences, and to replace the current practice of directly citing foreign guidelines. On this basis, differentiated intervention programs should be designed for populations in different regions and age groups. During the formulation of guidelines and verification of programs, it is necessary to systematically control and evaluate the impact of confounding factors (such as family habits, living environments, and academic pressure) and to verify their health benefits through localized empirical studies, to ensure the scientificity and applicability of the guidelines and intervention measures.

### 5.4 Explore multi-level influencing factors and focus on localized context to fill core research gaps

Existing research has prominent deficiencies in exploring the influencing factors of 24-h movement behaviours among Chinese children and adolescents. Internationally, a wealth of mature studies have been conducted on the multi-level influencing factors (individual, family, school, and societal) of 24-h movement behaviours (41–43), with family and parental factors (e.g., family activity environment, parental movement behaviour compliance, parent–child co-participation in physical activity, and parental attitudes towards screen time) widely recognized as key determinants that directly shape children and adolescents’ movement patterns (44–46). However, relevant domestic research in this field is still in the initial stage: most of the studies included in this review focus on the prevalence of movement behaviours and their association with health outcomes, with extremely scarce original empirical evidence targeting the influencing factors of these behaviours—especially the independent and interactive effects of family and parental factors (35). As a result, systematic integrated analysis and in-depth discussion on this topic are not feasible, which constitutes a core research gap that needs urgent attention in the domestic field.

Notably, the included studies have extremely limited empirical exploration on the association between typical Chinese social context factors and 24-h movement behaviours—such as the “Double Reduction” policy, the development of school physical education, and the widespread popularization of electronic products. Specifically, existing studies have not yet explored the specific impacts of the “Double Reduction” policy (4), for example, whether the increased after-school services have effectively changed the time allocation of physical activity, sedentary behaviour, and sleep, or how family supervision and parental participation interact with the policy to influence children’s movement behaviours (47). This represents a critical localized research gap, as the policy-induced time reallocation may fundamentally reshape children’s daily movement patterns but remains unexamined in current literature, and the synergy between family factors and policy implementation has not been explored.

Subsequent targeted original studies should be carried out to supplement high-quality evidence: on the one hand, to systematically investigate the multi-level influencing mechanisms of family environment, parental movement behaviours, parent–child interaction, and other family-related factors on children’s 24-h movement behaviours, clarifying how parental role models, family activity norms, and parent–child sports participation affect physical activity, sedentary time, and sleep quality; on the other hand, to clarify the complex effects of localized social context factors and their interaction with family factors.

## 6 Conclusion

This scoping review systematically summarizes the current status of research on 24-h movement behaviours of Chinese children and adolescents since 2019, offering a new perspective for subsequent research and policy formulation. Currently, multiple aspects of research on 24-h movement behaviours among Chinese children and adolescents require urgent improvement, particularly in terms of regional coverage, study design, and monitoring methods. It is necessary further to optimize research ideas and methods in this field, systematically analyze the current status of 24-h movement behaviours among Chinese children and adolescents, and then formulate laws, regulations, and behavioural guidelines tailored to the actual conditions of children and adolescents across different regions and with diverse characteristics in China.

## Data Availability

Publicly available datasets were analyzed in this study. This data can be found at: all datasets analyzed in this systematic review are derived from previously published primary studies. The corresponding data repository links, accession numbers, and availability details for each dataset are provided in the reference list of the included original studies, which are cited throughout this review.
