# Supplementary material for: A scoping review of 24-h movement behaviours research in Chinese children and adolescents
Source: Front Public Health. 2026 May 12;14:1801708. doi: 10.3389/fpubh.2026.1801708 (PMC13201467; doi:10.3389/fpubh.2026.1801708)
Supplement: Supplementary file 1 [file Table_1.DOCX]

**Additional file 1: Search strategy**

| Database | Search strategy | Retriever | Search date | Search result |
| --- | --- | --- | --- | --- |
| Web of Science | 24-h* Movement Behavio* (Abstract) or 24-h* Activity Behavio* (Abstract) or Sleep* (Abstract) or Sedentary (Abstract) or Physical Activity (Abstract) and Children (Abstract) or adolescent (Abstract) and China (Abstract) or Chinese (Abstract) | Zhang Feng | 2025.11.1 | 3119 |
| EBSCO | 24-h* Movement Behavio* (Abstract) or 24-h* Activity Behavio* (Abstract) or Sleep* (Abstract) or Sedentary (Abstract) or Physical Activity (Abstract) and Children (Abstract) or adolescent (Abstract) and China (Abstract) or Chinese (Abstract) | Zhang Feng | 2025.11.1 | 2031 |
| PubMed | ((24-h* Movement Behavio*[Title/Abstract] OR 24-h* Activity Behavio*[Title/Abstract]OR Sleep*[Title/Abstract] OR Sedentary[Title/Abstract] OR Physical Activity[Title/Abstract]) AND (Children[Title/Abstract] OR adolescent[Title/Abstract])) AND (China[Title/Abstract] OR Chinese[Title/Abstract]) | Zhang Feng | 2025.11.1 | 1453 |
| CNKI | (AB='24小时活动行为' OR AB='24小时运动' OR AB='睡眠时间' OR AB='久坐时间' OR AB='身体活动') AND (AB='儿童青少年' OR AB='儿童' OR AB='青少年') | Zhang Feng | 2025.11.1 | 1205 |
