# Supplementary material for: A scoping review of 24-h movement behaviours research in Chinese children and adolescents
Source: Front Public Health. 2026 May 12;14:1801708. doi: 10.3389/fpubh.2026.1801708 (PMC13201467; doi:10.3389/fpubh.2026.1801708)
Supplement: Supplementary file 3 [file Table_3.DOCX]

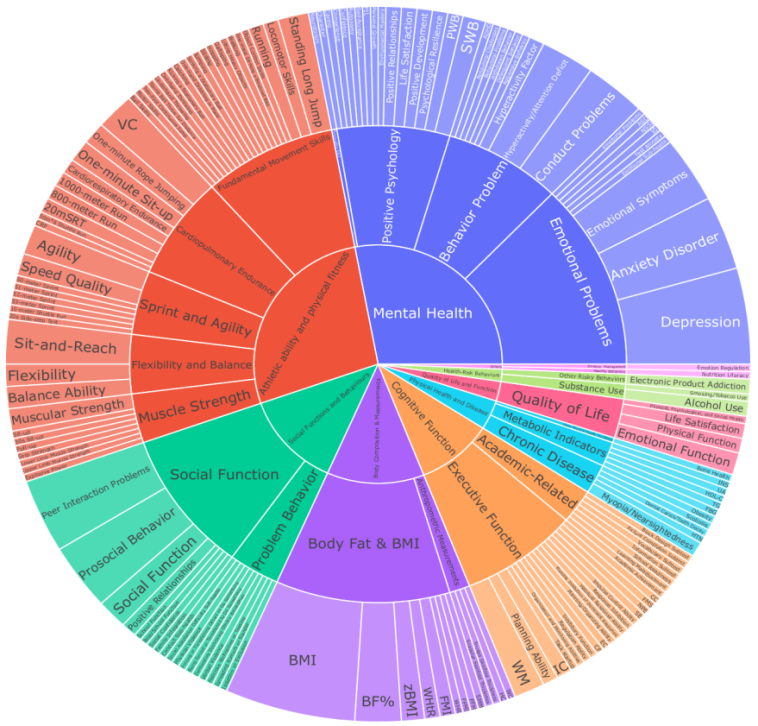


**Additional file 5: Sunburst chart of research variable hierarchies**

**NOTE:** BMI：Body Mass Index；BF%：Body Fat Percentage；zBMI：Body Mass Index z-score；WHtR: Waist-to-Height Ratio；WHR：Waist-to-Hip Ratio；FMI：Fat Mass Index；FFMI：Fat-Free Mass Index；FFM：Fat-Free Mass；SMM：Skeletal Muscle Mass；HC：Head Circumference；WC：Waist Circumference；SI：Suicidal Ideation；NSSI：Non-Suicidal Self-Injury；ASD：Autism Spectrum Disorder；ADHD：Attention-Deficit/Hyperactivity Disorder；SWB：Subjective Well-being；PWB：Psychological Well-being；PIL：Purpose in Life；VC：Vital Capacity；20mSRT：20-meter Shuttle Run Test；CRF：Cardiorespiratory Fitness；WM：Working Memory；IC：Inhibitory Control；CF：Cognitive Flexibility；EC：Emotional Control；SE：Switching Efficiency；NPE：Non-Perseverative Errors；FMS：Failure to Maintain Set；CC：Categories Completed；HTN：Hypertension；FBG：Fasting Blood Glucose；TG：Triglycerides；HDL-C：High-Density Lipoprotein Cholesterol；UA：Uric Acid；INS：Insulin
